# Historical Sketch of the Progress of Dental Science

**Published:** 1839-09

**Authors:** Harvey Burdell

**Affiliations:** Author of "Observations on the Anatomy, Structure, Physiology, and Diseases of the Teeth."


### HISTORICAL SKETCH

*Of the progress of Dental Science,—Translated from the French, with notes and general observations,*

[ BY HARVEY BURDELL, M. D. ]

Author of "Observations on the Anatomy, Structure, Physiology, and Diseases of the Teeth."—*(Continued.)*

### Third Period.

*From Galen to Versalia.*

Galen,* availing himself of the anatomical knowledge of his predecessors, but more particularly of the Alexandrians, was enabled to describe the teeth with greater advantage, and with more exactness than had been done previous to his time. He says the teeth are all formed during gestation; that the molares of the upper jaw have three roots, and those of the lower only two; that the canines are termed eye teeth, οφθαλμιχοι because they receive branches from the same nerve that extends to the eye. He was of the opinion that the teeth were REAL BONES: in ossium numero dentes habendi sunt, etsi secus nonnulli sophistæ arbitrentur. ***

*Albucassis*, was the first who asserted that the teeth could be transplanted,† or taken from the jaws of one person and placed into those of another, and yet maintain their living principle. I will, however, notwithstanding the momentary digression, mention a case related by Ambrosie Paré. We will allow him to relate the circumstance himself: "A princess was obliged to have a diseased tooth extracted, and on applying to a surgeon, she brought with her one of her waiting maids, who stood by the side of her mistress, and when the tooth of the princess had been extracted, a sound one also was taken from the jaw of the girl and placed into the socket of the jaw of the former, which took root and became sound and healthy."***

### Fourth Period.

*From Versalia to Harvey.*

Versalia succeeded in reclaiming the science of anatomy from the degraded condition into which it had fallen. He, however, appears to have

* Galen commenced the practice of his profession at Rome about the year 160, and on account of the success he met with in practice, his opponents declared that he was leagued with a super-human power, and thus "traduced him as a magician." He wrote more than 700 essays on different subjects, most of which were on medicine and anatomy. He died at the age of 70. H. B.

† The doctrine of transplanting teeth, so as to render them as healthy as the orignal ones is an absurd theory, for it is impossible, and contrary to the laws of physical science, to revive a living principle between the nerve of a tooth thus transplanted and the jaw bone, and it is doubted whether a healthy tooth can be extracted and replaced again, into the socket of the jaw of the same person, without destroying its vitality, although the celebrated Hunter was of a different opinion. H. B.

neglected the study of the anatomy and physiology of the teeth, and therefore has left no writings on the subject worthy of note. He says, however, that the teeth are similar to other bones, but that they differ from them because they are exposed to the action of the atmosphere ; and he also believed that the temporary or infant teeth assist in the formation of the second set.

*Eustachi* has given a much better and a more perfect description of the teeth than had been done by his predecessors.* He describes their anatomical structure, their shape, uses, number, and the diseases to which they are liable, in such a perfect manner as to leave but little unsaid respecting them. His views, however, in reference to their attachment to the jaws, may be considered hypothetical. ***** He coincides with some of his predecessors, that the hardness of the teeth of animals is in proportion to their ferocity. He distinguishes the bony portions of the teeth from the enamel, and compares the latter to the bark of trees. In a well written dissertation on the developement of the teeth, he gives a detailed sketch of the dental follicules, of their vessels, &c. The rudiments of both the first and second set, he says, exists in the fœtus. He says, also, that the teeth are supplied with a living principle, but that they are nourished differently from the bones, and he gives as a reason in proof of this position, that the teeth do not unite or become consolidated when broken, as is the case with the bones. He believes the teeth derive their sensibility from the nerves given off from the follicule, and that the tooth-ache arises from a pressure upon these nerves, or that the nerves swell and press against the bony parts of the teeth ; but this, he even admits to be merely a conjecture : *Ego quamquam certam demonstrationem non habeo, conjectura nihilominus adducor, et suspicer nervum, qui in concavitate dentium penetrat, in minutissimos surculos diffusum cum intima ipsorum substantia, quœ mucosa est initio generationis commisceri.*

### Fifth Period.

*From Harvey to Bichat.*

The study of the anatomy of the teeth during this intervening period, appears to have occupied the attention of professional men. The distinct essays that have appeared on the subject are very numerous, but as the science has gained so little from them, I shall not notice them in detail.

Becker, Schroeder, Bartholine and Genga, have written gross absurdities respecting the teeth. Bartholine mentions a case in which a single tooth occupied the whole circle of the jaw, and Genga relates a case of the growth of an *iron* tooth.

Whilst I am on the subject, I will mention the case of the golden tooth, which in former times produced so much *sensation* throughout the community. Several dental authors believed this absurd story. Ungebaur, however, very justly ridicules those credulous persons, who are ready to

---

* Eustachi was one of the most celebrated anatomists of the 16th century.—He was the first who gave a correct description of the renal capsules and the thoracic duct, and he also discovered a valve in the heart and an opening between the throat and internal ear. H. B.

believe whatever appears strange, and which they cannot directly comprehend. He, however, admits that many errors have been committed in reference to the composition of the teeth, from the fact, that they *often change their color*, and become yellow, or black, as the case may be, from concretions of foreign matter upon their surfaces. He also says, that the teeth of many ruminating animals take the color of the juice or sap of the plants upon which they may have fed.

*Fulchius*, in his dissertation, explains the fallacy of the golden tooth.—"Rhumbaumius," says he "saw a child, that it was pretended, had a golden tooth." This child was exhibited for money, as a great curiosity. Rhumbaumius evinced some doubt as to the actual composition of the tooth, and therefore sent a gold-smith and directed him to cut a small portion from it for the purpose of analysis. This was done, and the goldsmith declared the tooth to be real gold. The next day, however, Rhumbaumius called again to see the child's tooth, and to his surprise, the notch made by the goldsmith had disappeared, which caused him to suspect that fraud had been practiced, and he again examined the tooth with greater care than before, and on passing a stillet between the gum and the tooth, succeeded without much effort, in detaching a plate or cap of gold, which had been closely fitted over the projecting surface of a natural tooth. * * * *

*Fredericus* wrote a dissertation entitled: "De dentium statu naturali et præter naturali," in which he displayed great erudition. He commences this thesis by giving a detailed sketch of the importance and dignity of the teeth: *dignitas dentium.* In certain parts of India, he says that the teeth were formerly so esteemed, that they were offered as sacrifices to the gods; and the ancients, seeing that the teeth remained perfect after the body had been long entombed, supposed they assisted in the final resurrection.

He compared the phenomena of dentition to the germination of grain, or the sprouting of seeds whilst yet enveloped in their capsules: Totus dens primum inclusus est folliculo seu membrana tenui ac pellucida, non secus ac granum in arista.

---

We have received several epistolatory communications from Dr. Caldwell of Va. now resident in Philadelphia, in relation to the re-publication of Dr. Flagg's article in the first number of this journal, one of which he desired us to publish in conformity with our custom of receiving articles from contributors. If this communication, which was in the form of a letter to the publishing committee, had contained any of the Doctor's arguments in support of the ridiculous pretension to a new *Ligamentum Dentis* unknown to the knife of any former demonstrator, and to the lancet of any other dentist, we should certainly have published the article with great pleasure; but as it contained nothing but personal vituperation directed towards us individually, we have filed it among the favors of *interesting* correspondents. Should Dr. Caldwell or any other person, furnish a well written article for the pages of this journal on any subject connected with the usefulness of our profession—we shall gladly insert it without delay, in case communications from our subscribers do not claim precedence.

